# Clinical Detection of Chronic Rhinosinusitis through Next-Generation Sequencing of the Oral Microbiota

**DOI:** 10.3390/microorganisms8060959

**Published:** 2020-06-26

**Authors:** Ben-Chih Yuan, Yao-Tsung Yeh, Ching-Chiang Lin, Cheng-Hsieh Huang, Hsueh-Chiao Liu, Chih-Po Chiang

**Affiliations:** 1 Department of Otorhinolaryngology, Fooyin University Hospital, Pingtung 92849, Taiwan; b698@fy.org.tw; 2 Department of Education and Research, Fooyin University Hospital, Pingtung 92849, Taiwan; glycosamine@yahoo.com.tw (Y.-T.Y.); x6053@ms25.hinet.net (C.-C.L.); 3 Department of Medical Laboratory Sciences and Biotechnology, Fooyin University, Kaohsiung 83102, Taiwan; 4 Aging and Disease Prevention Research Center, Fooyin University, Kaohsiung 83102, Taiwan; 5 Program in Environmental and Occupational Medicine, Kaohsiung Medical University, Kaohsiung 80708, Taiwan; prevailingkimo@gmail.com; 6 Department of Laboratory Medicine, Fooyin University Hospital, Pingtung 92849, Taiwan; 9341@fy.org.tw; 7 Department of Surgery, Kaohsiung Medical University Hospital, Kaohsiung 80756, Taiwan; 8 Division of Breast Surgery, Department of Surgery, Kaohsiung Medical University Hospital, Kaohsiung 80756, Taiwan

**Keywords:** chronic rhinosinusitis (CRS), nasal microbiome, oral microbiome, saliva, next-generation sequencing (NGS)

## Abstract

Chronic rhinosinusitis (CRS) is the chronic inflammation of the sinus cavities of the upper respiratory tract, which can be caused by a disrupted microbiome. However, the role of the oral microbiome in CRS is not well understood. Polymicrobial and anaerobic infections of CRS frequently increased the difficulty of cultured and antibiotic therapy. This study aimed to elucidate the patterns and clinical feasibility of the oral microbiome in CRS diagnosis. Matched saliva and nasal swabs were collected from 18 CRS patients and 37 saliva specimens from normal volunteers were collected for 16S rRNA sequencing. The α-diversity of the saliva displayed no significant difference between control and CRS patients, whereas the β-diversity was significantly different (*p* = 0.004). Taxonomic indices demonstrated that *Veillonella dispar*, *Rothia mucilaginosa*, and *Porphyromonas endodontalis* were enriched, while *Campylobacter* and *Cardiobacterium* were reduced in the saliva of CRS patients. These microbial markers could significantly distinguish CRS patients from control (AUC = 0.939). It is noted that the 16S rRNA results of the nasal swab were consistent with the nasopharynx aerobic culture, and additionally detected multiple pathogens in CRS patients. In summary, these results indicated these oral microbiomes may provide a novel signal for CRS detection and that NGS may be an alternative approach for CRS diagnosis.

## 1. Introduction

Chronic rhinosinusitis (CRS) is a common upper respiratory tract disease, defined as a persistent inflammation of the nasal cavity and sinus mucosa for more than 12 weeks. CRS is frequently caused by viral and bacterial infection, resulting in symptoms of nasal congestion/discharge, facial pain/pressure, and loss of smell. These symptoms not only severely impact the patient’s quality of life and work ability but also cause an enormous economic burden [1,2]. According to whether nasal polyps are present, CRS is further divided into chronic rhinosinusitis with nasal polyposis (CRSwNP) and chronic rhinosinusitis without nasal polyposis (CRSsNP). When the number of polyps is too many or they become too large, they will further block the nasal cavity, preventing normal mucus discharge and worsening the infection. Until now, the therapeutic approach for CRS has been treatment with antibiotics, corticosteroids, saline lavage, and surgery [3].

CRS pathogenesis is a complex process of microbial infection and inflammation. Numerous studies have investigated the microbiome in the sinuses of normal subjects and CRS patients [4,5]. However, few studies have reported the patterns and clinical relevance of oral microbiomes in CRS patients. The oral cavity and neighboring nasal cavity are ideal habitats for microbiomes due to the stable oral temperature, pH, and nutrient transportation from saliva [6,7]. It is noted that the oral microbiome is the second most complex microbial system behind the gut microbiota. Approximately 700 species have been reported in the oral cavity; half of them are cultivated and named [8]. The oral cavity is also an important entrance for both the upper and lower respiratory tracts. Oral pathogens have been reported to impact systemic health through the bloodstream, swallowing, or other avenues and are involved in systemic diseases, especially for periodontal disease in cardiovascular disease, diabetes, and Alzheimer’s disease [9,10,11].

The clinical characteristics of CRS are a polymicrobial infection and an increased proportion of anaerobes. The polymicrobial infection increases the difficulty of antibiotic therapy, and most of the anaerobic species are uncultivated [4]. Therefore, use of the culture-independent molecular approach has been growing in the past few years with pyrosequencing [12,13], quantitative polymerase chain reaction (qPCR) [14], and next-generation sequencing (NGS) [15]. However, few studies have assessed different sites of the microbiome in the same CRS patients, especially within the oral microbiome in CRS. Furthermore, conflicting results exist between culture-dependent and culture-independent approaches. This study aimed to investigate the patterns of the oral microbiome in CRS patients, providing a non-invasive approach for CRS detection with saliva. Additionally, we compared the results of clinical nasopharynx aerobic culture with the NGS results of nasal swabs from the same CRS patients, allowing us to explore the clinical feasibility of comprehensive microbiome analysis using NGS in CRS patients.

## 2. Materials and Methods

### 2.1. Patient Recruitment

In total, 55 subjects were recruited, including 37 normal control volunteers and 18 chronic rhinosinusitis (CRS) patients. In the control group, the saliva specimens were collected by flow saliva into a 50-mL centrifuge tube containing storage buffer and stored at 4 °C for DNA extraction. In CRS patients, the specimen was collected from the same patient in multiple sites, including a saliva (SINS) and nasal swab (SINNS). The saliva specimen was collected by the above approach and a nasal swab was obtained by a clinician. All the specimens were collected before treatment with antibiotics and drugs, and all the subjects had approval from the Internal Review Board and informed consent from all subjects (FYH-IRB-107-04-02, 17 August 2018).

### 2.2. Saliva and Nasal Swab of DNA Extraction

Saliva and nasal swab specimens were collected and stored at 4 °C for DNA extraction. Genomic DNA was extracted with the QIAamp BiOstic Bacteremia DNA Kit (Qiagen, Germantown, MD, USA) according to the manufacturer’s instructions. Briefly, samples were centrifuged at 10,000 rpm for 10 min to remove storage buffer and lysed with MBL solution and homogenized with FastPrep-24 5G (MP biomedicals, Irvine, CA, USA). Finally, the supernatant was washed with the MB spin column and eluted with the EB solution. The concentration was assessed with a NanoDrop 2000 spectrophotometer.

### 2.3. PCR Amplification and 16S Sequencing

The library was constructed with the standard V3–V4 region of the 16S rRNA gene. PCR was amplified with KAPA HiFi hotstart readymix (Roche, Branchburg, NJ, USA) and following the instructions of Illumina 16S metagenomics sequencing library preparation. The PCR product was further purified with AMPure XP magnetic beads (Beckman Coulter, Brea, IN, USA) and barcoded by the Nextera XT index kit (Illumina, San Diego, CA, USA). The PCR product’s amplification and quality were assessed by a Fragment Analyzer (Advanced Analytical, Ankeny, IA, USA) and quantified by the Qubit dsDNA HS assay kit (Life Technologies, Pleasanton, CA, USA). In total, 20% of PhiX control was added into the final pool to 10 pM and the library was sequenced on a MiSeq (Illumina, San Diego, CA, USA) with the paired-end reads (2 × 300 nt) using a MiSeq Reagent Kit V3 600 cycles. Approximately 800 (K/mm^2^) clusters were generated and over the 90% passing filter with Q30 ≥ 80%, with at least >50,000 reads per sample. FASTQ files were collected and used for further analysis.

### 2.4. Bioinformatics Analysis

The raw paired-end reads were trimmed and passed through quality filters (quality trimming, discarding short read length, and removing chimeras), and were assigned to operational taxonomic units (OTUs), which share ≥ 97% similarity with the Greengene database. The raw paired-end reads were also analyzed with the basespace Ribosomal Database Project (RDP) classifier. OTU taxonomic (relative abundance, heatmap, Krona, differential abundance analysis), α-diversity (Shannon index), and β-diversity (PCoA-Weighted UniFrac) were performed with basespace (Illumina, San Diego, CA, USA), CLC Microbial Genomics Module (Qiagen, Germantown, MD, USA), and GraphPad prism 7 (GraphPad Software, La Jolla, CA, USA). The OTU table was generated by the CLC Microbial Genomics Module to be further analyzed with the linear discriminant analysis effect size (LEfSe) and Phylogenetic Investigation of Communities by Reconstruction of Unobserved States (PICRUSt) analysis. LEfSe was performed by Galaxy/HutLab to identify specific microbial markers between groups with an alpha value for the factorial Kruskal–Wallis test/pairwise Wilcoxon test of 0.01 and LDA score cut-off of 2.0. PICRUSt prediction was performed by Galaxy/HutLab according to the Kyoto Encyclopedia of Genes and Genomes (KEGG) functional pathways database and analyzed with Statistical Analysis of Metagenomic Profiles (STAMP) software. The STAMP criteria were set up with removing unclassified reads, *p* < 0.01, and effect size of 0.5. The results revealed those functional pathways with a significantly different abundance at level 3 between groups. A comparison of different groups was performed by the two-tailed t-test. A *p* value less than ★ *p* < 0.05, ★★ *p* < 0.01, ★★★ *p* < 0.001, and ★★★★ *p* < 0.0001 was considered statistically significant. The specificity and sensitivity of the microbial marker were analyzed with the receiver operating characteristic curve (ROC curve) and the area under curve (AUC) value.

## 3. Results

### 3.1. CRS Patients Had a Lower Diversity of the Nasal Microbiome

**Figure 1:**
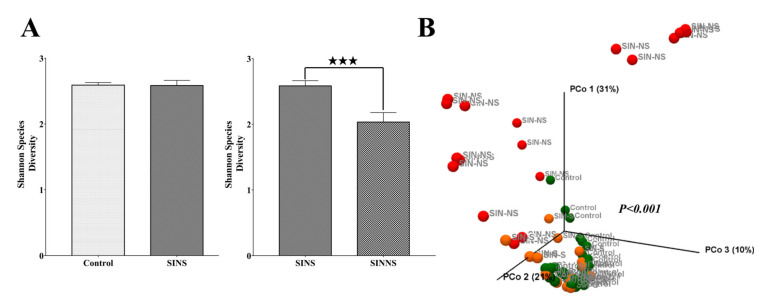
The microbial diversity of different subjects. (**A**) The α-diversity of the Shannon index was lower in the nasal swabs of CRS patients (SINNS) than the saliva of CRS patients (SINS); (**B**) The β-diversity of PCoA demonstrated a significant difference in the total microbial composition among the control, SINS, and SINNS. A *p* value less than ★ *p* < 0.05, ★★ *p* < 0.01, ★★★ *p* < 0.001, and ★★★★ *p* < 0.0001 was considered statistically significant.

Fifty-five subjects were divided into three groups, including the control: Saliva from normal volunteers; SINS: saliva from CRS patients; and SINNS: nasal swab from the same CRS patients. We analyzed these three groups with the α/β-diversity initially. The Shannon index, which indicates the diversity of microbiome communities, was calculated to assess the α-diversity of these three groups. The α-diversity displayed no significant difference between the control and CRS patients in saliva (SINS). In contrast, the diversity in the nasal microbiome of CRS patients (SINNS) was significantly lower than the saliva of CRS patients (Figure 1A).

The β-diversity with the principal coordinate analysis (PCoA) of Weighted-UniFrac was assessed to evaluate the microbial composition among the control, SINS, and SINNS groups. The PERMANOVA test indicated a significant difference (*p* < 0.001) of the overall microbial composition among these three groups (control versus SINS: *p* = 0.004; control versus SINNS: *p* < 0.001; SINS versus SINNS: *p* < 0.001, Figure 1B). Thus, the diversity in the nasal microbiome of CRS patients was lower than in the saliva of the same patient. Meanwhile, the total microbial composition was different among these three groups.

### 3.2. Operational Taxonomic Units (OTUs) of the Phylum and Species Level among Different Groups

**Figure 2:**
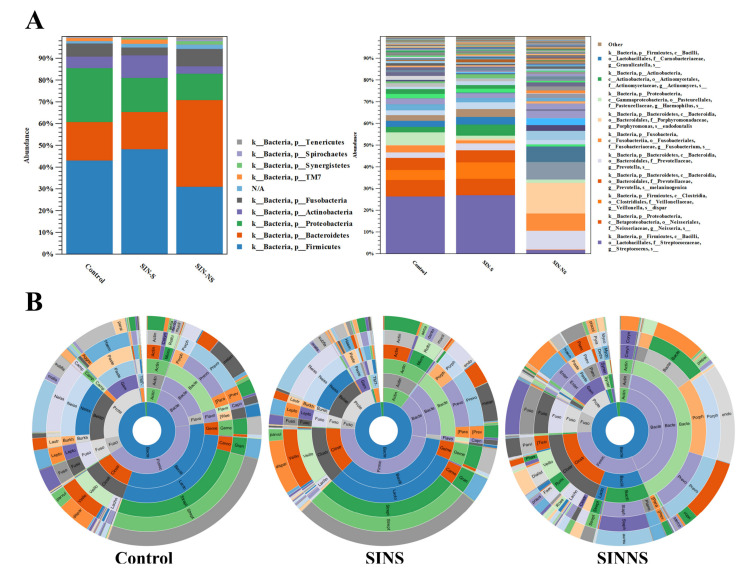
The relative abundance of OTUs among different groups. (**A**) The relative abundance of OTUs at the phylum and species level; (**B**) Krona analysis revealed that the relative abundance of *Veillonella dispar* and *Porphyromonas endodontalis* were enriched in the multiple-site samples of saliva (SINS) and nasal (SINNS) from CRS patients.

The β-diversity with PCoA indicated that the total microbial composition was different among the three groups. Thus, we further investigated different microbial levels based on the OTUs. At the phylum level, most OTUs was *Firmicutes* in both the saliva of the control and SINS, whereas the abundance of *Bacteroidetes* was higher in the nasal swabs of SINNS. At the species level, *Veillonella dispar* and *Porphyromonas endodontalis* were more abundant in both the saliva and nasal swabs from CRS patients (Figure 2A). This phenomenon was more obviously seen with the Krona analysis, where *Veillonella dispar* and *Porphyromonas endodontalis* were enriched in both the saliva and nasal swabs from CRS patients compared with the saliva from the control (Figure 2B).

### 3.3. Specific Microbial Markers in CRS Patients

**Figure 3:**
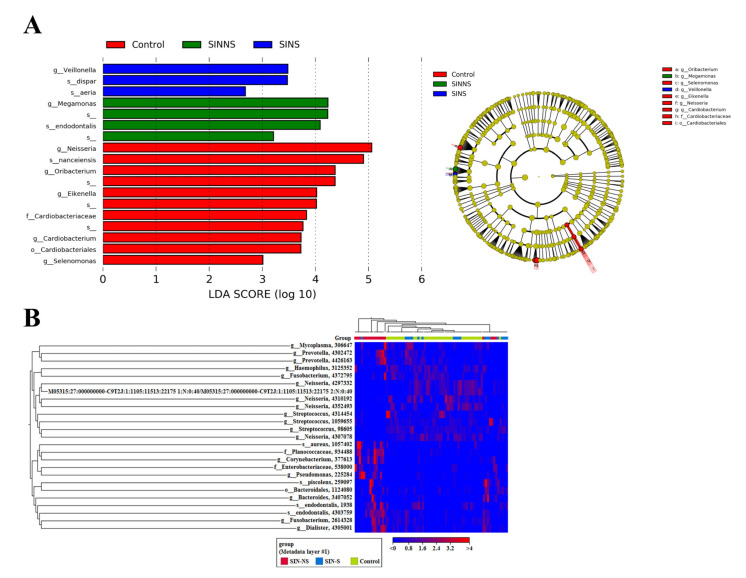
Microbial markers in the different specimens of CRS patients. (**A**) LEfSe analysis revealed that specific microbial markers, including *Veillonella dispar* in the saliva, and *Porphyromonas endodontalis* in the nasal swabs of CRS patients; (**B**) Heatmap analysis indicated the abundance of the microbiome as high (red) and low (blue) regarding these three groups.

One of this study’s goals was to investigate microbial markers from the non-invasive saliva of CRS patients. Therefore, we further clarified the microbial markers based on the linear discriminant analysis effect size (LEfSe) and OTU taxonomic of heatmap analysis. An LEfSe analysis based on an LDA score > 2, alpha value for the factorial Kruskal–Wallis test/pairwise Wilcoxon test of 0.01 was conducted and concentrated on the genus/species level. The CRS patients (SINS)’ saliva was enriched with *Veillonella dispar* and *Rothia aeria*, whereas several genera like *Neisseria* and *Cardiobacterium* were present in the controls’ saliva. *Megamonas* and *Porphyromonas endodontalis* were more abundant in the nasal microbiome of CRS patients (SINNS) (Figure 3A).

This result was similar to previous OTUs, and we also used heatmap to assess the richness of several genus/species as high (red) and low (blue) regarding these three groups (Figure 3B). These results indicated that several potential microbial markers, such as *Veillonella dispar* and *Porphyromonas endodontalis*, coexisted in the different specimens of CRS patients.

### 3.4. A Novel Microbial Ratio from Saliva to Distinguish CRS Patients from the Control Group

**Figure 4:**
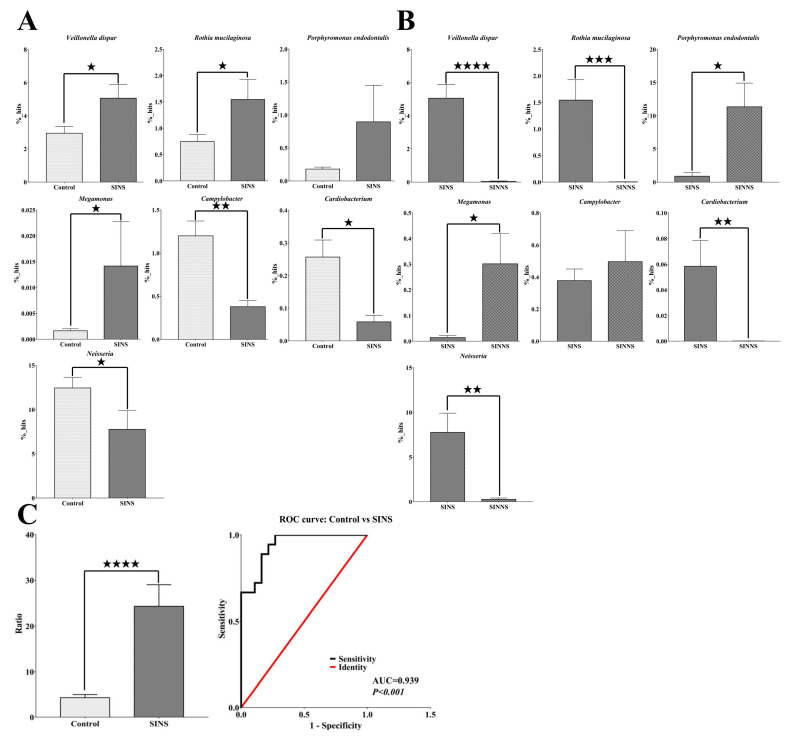
A non-invasive, oral, and saliva-based microbial marker for the diagnosis of CRS patients. (**A**,**B**) Several genus/species from the LEfSe/heatmap/differential abundance analysis were further verified by RDP classification, showing that *Veillonella dispar* and *Rothia mucilaginosa* were significantly higher in the saliva of CRS patients and that *Porphyromonas endodontalis* was enriched and coexisted in the multiple sites of saliva and nasal swabs of the same CRS patients; (**C**) The average ratio derived from the sum of percentages of the increased microbial markers (*Veillonella dispar*, *Rothia mucilaginosa*, and *Porphyromonas endodontalis*) divided by the sum of percentages of the lower (*Campylobacter* and *Cardiobacterium*) microbial markers provided outstanding discrimination (AUC = 0.939) for identifying CRS patients from the control. A *p* value less than ★ *p* < 0.05, ★★ *p* < 0.01, ★★★ *p* < 0.001, and ★★★★ *p* < 0.0001 was considered statistically significant.

To further clarify microbial markers and elucidate which pathogenic species coexisted in both the saliva and nasal samples of CRS patients, we analyzed all of the potential genus/species microbiomes from LEfSe, heatmap, and differential abundance analysis. These potential genus/species microbiomes were reanalyzed by RDP classifier and eliminate the microbiomes with lower percentage or no statistically significant in CRS patients. Eventually, we identified seven bacterial taxa that were statistically significant in CRS patients. Compared with the controls’ saliva, *Veillonella dispar* (control: 2.94%; SINS: 5.05%, *p* = 0.013) and *Rothia mucilaginosa* (control: 0.75%; SINS: 1.54%, *p* = 0.019) were specific oral saliva microbial markers that were significantly higher in CRS patients. *Megamonas* was higher in saliva (control: 0.001%; SINS: 0.014%, *p* = 0.04) and nasal (SINS: 0.014%; SINNS: 0.3%, *p* = 0.02) samples of CRS patients. Most importantly, we identified *Porphyromonas endodontalis,* a pathogenic species that was enriched and coexisted in multiple sites of saliva (control: 0.18%; SINS: 0.9%, *p* = 0.07) and nasal (SINS: 0.9%; SINNS: 11.35%, *p* = 0.01) samples from the same CRS patients. In contrast, *Campylobacter* (control: 1.2%; SINS: 0.38%, *p* = 0.002), *Cardiobacterium* (control: 0.26%; SINS: 0.06%, *p* = 0.01), and *Neisseria* (control: 12.44%; SINS: 7.75%, *p* = 0.04) were significantly lower in both the saliva and nasal samples of CRS patients (Figure 4A,B).

We further defined a ratio of five microbial markers of the saliva from the sum of percentages of the increased microbial markers (*Veillonella dispar*, *Rothia mucilaginosa*, and *Porphyromonas endodontalis*) divided by the sum of percentages of the decreased (*Campylobacter* and *Cardiobacterium*) microbial markers. This average ratio (control: 4.37 versus SINS: 24.32, *p* < 0.0001) was further analyzed by the receiver operating characteristic curve (ROC curve). The area under the curve (AUC) was 0.939 (*p* < 0.0001), displaying outstanding discrimination for distinguishing CRS patients from the control. This indicated that the ratio was a potential microbial index from a non-invasive saliva specimen that could diagnose CRS from a normal control (Figure 4C).

### 3.5. Microbiome-Related Functional Pathways in Chronic Rhinosinusitis

**Figure 5:**
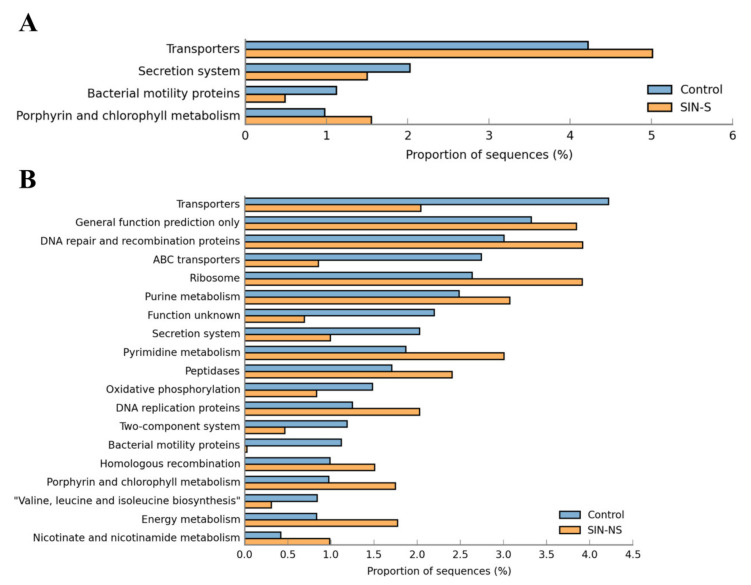
Microbiome-related functional pathways in the saliva and nasal swabs of CRS patients. (**A**) Functional pathways of transporters, porphyrin, and chlorophyll metabolism were increased, while secretion systems and bacterial motility proteins were lower in the SINS-related microbiome; (**B**) A more complex and pronounced effect of several functional pathways was observed in the SINNS-related microbiome.

To further realize the microbiome’s function in CRS patients, analysis against KEGG level 3 pathways using the microbial markers from LEfSe was conducted. After filtering with the criteria of the removed unclassified reads, *p* < 0.01, and an effect size of 0.5 from STAMP software, there were four functional pathways enriched in the control versus SINS and 19 functional pathways enriched in the control versus SINNS. Compared with the control, the abundance of the microbiome in SINS was increased in the functional pathways of transporters, porphyrin, and chlorophyll metabolism, and reduced in secretion systems and bacterial motility proteins (Figure 5A). Whereas in the SINNS microbiomes, there were more complex and significant results on functional pathways, including increases in porphyrin and chlorophyll metabolism, energy metabolism, nicotinate, and nicotinamide metabolism, and a reduction of transporters, ABC transporters, bacterial motility proteins, and secretion systems (Figure 5B). These results indicated a link and overlapping functional pathways and microbiomes between the oral and nasal cavity.

### 3.6. Comparison of Clinical Aerobic Culture and NGS Approaches

In addition to identifying oral microbial markers of CRS patients, another goal of this study was to compare culture-based methods with NGS in pathogen identification. The results are summarized in Table 1, with a total of 18 CRS patients (SINNS1-18). We collected the medical records of their nasopharynx aerobic cultures, which were routinely examined, and the results are listed in the upper part of the table with common aerobic culture. Culture examination identified *Staphylococcus aureus* from SINNS1, *Pseudomonas aeruginosa* from SINNS5, *Staphylococcus pneumoniae* from SINNS11, *Haemophilus influenza* from SINNS14, and other patients showed no isolated beta *Streptococcus* group A. The NGS results were entirely consistent with the culture-based pathogen identification showing SINNS1 with 62.43% of *Staphylococcus aureus*, SINNS5 with 48.81% of *Pseudomonas aeruginosa*, SINNS11 with 0.268% of *Staphylococcus pneumoniae*, and SINNS14 with 2.59% of *Haemophilus influenza*.

Additionally, we listed several important pathogens of CRS according to the aerobes and anaerobes of periodontopathogens from the RDP classifier of the NGS results. From Table 1, the NGS results indicated that culture-based methods missed many important pathogenic species that NGS identified, such as SINNS6 had 21.003% of *Haemophilus influenza* and SINNS18 had 40.028% of *Staphylococcus aureus*. Apart from the aerobic pathogens identified, most CRS patients also have multiple pathogenic infections with anaerobes, which require anaerobic culture in the laboratory. These anaerobic infections included *Porphyromonas endodontalis*, *Fusobacterium nucleatum*, and *Prevotella oris*. In the list of anaerobes, the most important anaerobic pathogen identified was *Porphyromonas endodontalis*, which is an important periodontopathogen that coexists abundantly in the saliva and nasal regions of CRS patients. Thus, these results suggest that the NGS-based platform might be a better approach to diagnose CRS patients due to its ability to simultaneously and massively detect aerobic and anaerobic pathogens.

## 4. Discussion

CRS is an inflammatory disease of the upper respiratory tract and is caused by a complicated infection related to the microbiome of the nasopharynx. Numerous studies have identified the pathogens associated with CRS, including *Staphylococcus aureus, Streptococcus pneumoniae, Streptococcus pyogenes. Haemophilus influenzae*, and *Moraxella catarrhalis*, which differ from healthy nasal flora [3,4]. However, reports of the oral microbiome in CRS are scarce.

In this study, we initially found that the α-diversity of the Shannon index was similar between the control and SINS. It is noted that an increased α-diversity of the gut microbiome may provide benefits to the healthy gut environment, and a loss of microbiota diversity (LOMD) is frequently connected to intestinal dysbiosis/disease [16,17]. As for the oral microbiome, α-diversity also decreases with disease progression [10]; however, in specific diseases, such as periodontitis, the diversity of the microbiome increases as the disease progresses [18,19]. Compared with the SINS, the α-diversity of SINNS was significantly lower. The diversity of the nasal cavity is affected by a classical bacterial infection, where diversity decreases with dysbiosis, an outgrowth of pathogen [18], and in most studies, CRS tends to reduce diversity [20,21]. In the β-diversity of the total microbial composition, there was a significant difference among the control, SINS, and SINNS (*p* < 0.001), suggesting that a different microbiome was present after disease development in the oral and nasal cavities.

To examine the differences in the total microbial composition and explore the oral microbial markers of CRS, we focused on the saliva from control and SINS samples and identified several oral microbial markers using LEfSe and heatmap analysis. The oral microbiome is a complex community with different sites of the microbiome, including saliva, soft tissue surfaces (oral mucosa and tongue), and hard tissue surfaces (teeth); the predominant genus is *Streptococcus,* which accounts for 20% of the oral microbiome [22]. Among these different sites of the oral microbiome, saliva is used as the most common approach to analyze the oral microbiome since it has similar microbial profiles to soft tissue, flows around the oral cavity, and can be sampled more easily [7,8]. Additionally, diagnostic markers in saliva are recognized for several oral diseases [23,24].

In our results, we found *Veillonella dispar* and *Rothia mucilaginosa* (*Rothia aeria* did not reach statistical significance) were significantly increased in the saliva of CRS patients. *Veillonella* spp. are anaerobic Gram-negative cocci that reside in the oral cavity and intestinal tract, and use lactate for fermentation [25,26]. Currently, there are 13 described species of *Veillonella* spp. that are reported predominantly in chronic periodontitis [27,28]; these can form lipopolysaccharides (LPSs) [29], and have antibiotic resistance [30,31], creating difficulties in the treatment of periodontitis. There is little knowledge concerning *Veillonella dispar* in infectious disease, with only a few cases reported, such as infective endocarditis [32,33], prosthetic joint infection [34], and chronic periodontitis [28]. *Rothia mucilaginosa* is an opportunistic pathogen that is an aerobic or facultative anaerobic and resides in the oral cavity and upper respiratory tract [35]. *Rothia mucilaginosa* infections are usually observed in immunocompromised patients [36,37] and are also involved in periodontal disease [38,39]. *Veillonella dispar* and *Rothia mucilaginosa* are both pathogens associated with periodontal disease in the oral cavity, suggesting that periodontal disease may contribute to CRS development.

We also identified *Porphyromonas endodontalis,* which was higher in both the saliva and nasal swabs of CRS patients. *Porphyromonas endodontalis* is a black-pigmented Gram-negative anaerobic oral pathogen species that is highly associated with chronic periodontitis, endodontic infections, and tooth pulp necrosis [40,41,42]. This black-pigmented bacteria (BPB) is frequently observed in infections of the oral cavity and respiratory tract. Thus, *Porphyromonas endodontalis* is an important anaerobic pathogen in CRS [4]. The coexistence of *Porphyromonas endodontalis* in the saliva and nasal swabs of CRS patients indicates that this oral pathogen might infiltrate into the nasopharynx. In contrast to increased levels of the oral pathogens, *Campylobacter* and *Cardiobacterium* were reduced in the saliva of CRS patients. *Campylobacter* commonly causes acute intestinal infection disease, such as diarrhea and vomiting, and is divided into two groups of species, zoonotic and oral. Zoonotic species are usually involved in gastrointestinal disease, and oral species are associated with periodontitis [43,44]. However, *Campylobacter* also accounts for 1% of the oral microbiome in the saliva of healthy adults [19] and this proportion was identical to our control group but lower in CRS patients. *Cardiobacterium* is a Gram-negative bacillus bacterium of the HACEK group (represents the species of *Haemophilus, Aggregatibacter,* *Cardiobacterium, Eikenella,* and *Kingella)* and accounts for 5–10% of infections in endocarditis patients who do not use intravenous drugs [45,46]. Currently, only two species of *Cardiobacterium hominis* and *Cardiobacterium valvarum* have been identified. In addition to rarely causing endocarditis, *Cardiobacterium* are also normally present in the oral and upper respiratory tract at less 1% [19,47], which is similar to our results, and also lower in CRS patients.

In our results, periodontal pathogen species were enriched frequently in the saliva of CRS patients. The oral microbiome has a significant impact, not only on oral diseases, such as caries and periodontitis, but also systemic diseases, including cancer, cardiovascular disease, and diabetes [48]. Additionally, these diseases are correlated with the periodontal pathogens of BPB (e.g., *Porphyromonas endodontalis*, *Porphyromonas gingivalis*, and *Prevotella intermedia*) and red complex bacteria (*Porphyromonas gingivalis*, *Treponema denticola*, and *Tannerella forsythia*) [49]. However, few studies have investigated the oral microbiome in CRS. Therefore, this is a novel study on the role of the oral microbiome, especially for periodontal pathogens in CRS. Periodontal disease has a 3.45-fold higher association with chronic maxillary sinusitis, and approximately 40% of sinusitis cases may have an underlying dental disease [50,51,52]; therefore, a possible clinical implication is that improved dental hygiene could prevent CRS.

Several studies have applied oral probiotics or topical probiotics in the prevention and treatment of CRS [53]. In contrast to antibiotics, probiotics can reverse the diversity of the sinus microbiome [54]. Oral probiotic administration is beneficial in treating CRS [55], reducing colonization of pathogenic species [56], and preventing upper respiratory tract infections [57]. These studies indicated the important roles of dental hygiene and oral microbiome in CRS. In addition to indicating a possible method of preventing CRS, we also investigated a non-invasive diagnostic approach using saliva to detect CRS with a ratio of five microbial markers from the sum of percentages of the increased microbial markers (*Veillonella dispar, Rothia mucilaginosa, Porphyromonas endodontalis*) divided by the sum of percentages of the decreased (*Campylobacter, Cardiobacterium*) microbial markers. This ratio displayed an outstanding means of distinguishing CRS patients from control (AUC = 0.939) and further expands the application of the saliva microbiome in disease diagnosis [58,59,60].

Based on the LEfSe analysis, the abundance of microbial markers was enriched in several functional pathways. Compared with the control, saliva had an increased abundance of the microbiome for transporter systems but was decreased for secretion systems. *Veillonella* spp. and *Rothia mucilaginosa* have been reported with the highest level of membrane transport systems in carious and periodontitis. This increase in transporter systems may affect the efficiency of transportation and therapy resistance [61,62], whereas the decrease of the secretion system may affect secreted protein exportation through the inner and outer membranes of Gram-negative bacteria [63]. Bacterial motility proteins consist of different protein complexes, which are responsible for the motility of bacterial species and are divided into two major groups of bacterial chemotaxis and flagella [64]. The reduced microbial abundance in the pathway of bacterial motility proteins may result from oral pathogen colonization and biofilm formation [65], whereas the increased microbial abundance in the pathway of porphyrin and chlorophyll metabolism may result from an increase in *Porphyromonas* spp. and *Veillonella* spp. [66,67]. These functional pathways were also identified in the nasal swabs of CRS patients, where they displayed more complex patterns than the control.

In addition to the oral microbiome in CRS, another clinical issue of CRS is the polymicrobial infection and an increased proportion of anaerobes. BPB, such as *Prevotella* and *Porphyromonas* spp., were identified as important anaerobic pathogens in CRS [4]; however, BPB are obligate anaerobes with similar biological characteristics, which render BPB difficult to cultivate and presents challenges for distinguishing BPB species based on the culture method in clinical detection [68,69], which is also a critical issue regarding polymicrobial infection. With the development of molecular techniques, polymerase chain reaction (PCR) and NGS have been applied to the detection of bacteria in CRS cases, and these techniques have had both consistent and conflicting results compared with culture-based methods [14,15,70,71]. However, in our results, the detection of pathogens by NGS was entirely consistent with clinical culture detection; NGS also detected pathogens that the culture methods failed to identify, and simultaneously detected polymicrobial infection, such as BPB species in CRS patients.

The present study has a number of limitations. The subject numbers in this study were relatively small, and more participants are required to solidify the ratio of oral microbial markers. However, our results points to new possibilities for future research on the oral microbiome and NGS for CRS prevention and clinical detection. Further research and clinical studies are required to explore and transfer the NGS-based platform to a fast screening platform using real-time PCR.

## 5. Conclusions

In conclusion, we conducted a novel study that investigated the oral microbiome in CRS and could diagnose the presence of CRS accurately in clinical subjects; additionally, these results indicated that CRS may be prevented using non-invasive testing of the saliva microbiome and improved dental hygiene. Pathogen identification by NGS was not only entirely consistent with clinical laboratory culture testing but was also able to detect polymicrobial and anaerobic infection simultaneously, indicating that the NGS-based platform could be a better approach in clinical detection of CRS.

## Figures and Tables

**Table 1:** Microbiome of chronic sinusitis detection from common aerobic culture and next-generation sequencing (NGS).

	SINNS1	SINNS2	SINNS3	SINNS4	SINNS5	SINNS6	SINNS7	SINNS8	SINNS9	SINNS10	SINNS11	SINNS12	SINNS13	SINNS14	SINNS15	SINNS16	SINNS17	SINNS18
**Common aerobic culture** **(Nasopharynx)**	Staphylococcus aureus	No beta Streptococcus group A	No beta Streptococcus group A	No beta Streptococcus group A	Pseudomonas aeruginosa	No beta Streptococcus group A	No beta Streptococcus group A	No beta Streptococcus group A	No beta Streptococcus group A	No beta Streptococcus group A	Staphylococcus pneumoniae	No beta Streptococcus group A	No beta Streptococcus group A	Haemophilus influenzae	No beta Streptococcus group A	No beta Streptococcus group A	No beta Streptococcus group A	No beta Streptococcus group A
**NGS**	62.43%				48.81%						0.268%			2.59%				
**NGS: Aerobes**																		
*Staphylococcus aureus*	**62.43%**	0%	0.006%	0%	0%	0%	0%	0.006%	0%	**0.341%**	0%	0.005%	0%	0%	0.003%	**0.105%**	0%	**40.028%**
*Streptococcus pyogenes*	0%	0%	0%	0%	0%	0%	0%	0%	0%	0%	0%	0%	0%	0%	0%	0%	0%	0%
*Streptococcus pneumoniae*	0%	0%	0%	0%	0%	**0.011%**	0%	0%	0%	0%	**0.268%**	0%	0.001%	0%	0%	0%	0%	0%
*Haemophilus influenza*	0%	0%	0%	0%	0%	**21.003%**	0%	0%	0%	**0.057%**	0%	0.001%	**0.01%**	**2.59%**	0%	0%	0%	0%
*Moraxella catarrhalis*	0%	0%	0%	0%	0%	0%	0%	0%	0.001%	0%	0%	0%	0%	0%	0%	0%	0%	0%
*Pseudomonas aeruginosa*	0%	0%	0%	0.001%	**48.81%**	0%	0.003%	0.003%	0%	**0.118%**	0%	0%	0%	0%	0%	**0.012%**	0.002%	**0.117%**
**NGS: Anaerobes-** **periodontopathogens**																		
*Porphyromonas gingivalis*	0%	0%	0%	0%	0.001% (Saliva: 2.246%)	0%	0%	**0.228% (Saliva: 0.598%)**	0%	**0.042% (Saliva: 0.322%)**	0%	0%	0%	**0.023% (Saliva: 0.246%)**	0.001% (Saliva: 0.472%)	0.001% (Saliva: 0%)	**0.2% (Saliva: 0.097%)**	0%
*Porphyromonas endodontalis*	0%	**3.352% (Saliva: 0.069%)**	**43.814% (Saliva: 0.039%)**	0.008% (Saliva: 0.012%)	0%	0.001% (Saliva: 0.14%)	0.002% (Saliva: 0.03%)	**2.33% (Saliva: 10.161%)**	**21.005% (Saliva:0.254 %)**	**44.197% (Saliva:0.355 %)**	0.003% (Saliva: 0.245%)	**11.3% (Saliva: 1.223%)**	**19.587% (Saliva:0.002 %)**	**15.89% (Saliva:0.834 %)**	**0.286% (Saliva:0.468%)**	**11.323% (Saliva: 0.856%)**	**31.165% (Saliva: 1.186%)**	0.007% (Saliva: 0.015%)
*Fusobacterium nucleatum*	0%	**1.57% (Saliva: 0.133%)**	**0.487% (Saliva: 0.089%)**	0%	0%	0%	0%	**0.183% (Saliva: 0.789%)**	**0.942% (Saliva: 0.031%)**	**1.151% (Saliva: 0.214%)**	0.005% (Saliva: 0.125%)	**9.509% (Saliva: 1.027%)**	**0.219% (Saliva: 0.026%)**	**7.014% (Saliva: 0.383%)**	0.001% (Saliva: 0.284%)	**11.012% (Saliva: 0.154%)**	**20.261% (Saliva: 2.068%)**	0%
*Prevotella oris*	0%	**27.092% (Saliva: 1.31%)**	**28.478% (Saliva: 0.15%)**	**0.014% (Saliva: 0.02%)**	0%	0%	0.001% (Saliva: 0.03%)	**0.986% (Saliva: 8.238%)**	0.004% (Saliva: 0.001%)	**11.349% (Saliva: 0.427%)**	0.003% (Saliva: 0.036%)	**6.93% (Saliva: 1.219%)**	0.004% (Saliva: 0.032%)	0.003% (Saliva: 0.019%)	**0.256% (Saliva: 0.03%)**	0.001% (Saliva: 0.025%)	0.001% (Saliva: 0.111%)	0.002% (Saliva: 0.262%)
*Prevotella intermedia*	0%	**0.159% (Saliva: 0.024%)**	**0.076% (Saliva: 0.022%)**	**0.014% (Saliva: 0.02%)**	0%	0%	0.001% (Saliva: 0.288%)	0%	0%	0%	0%	0.001% (Saliva: 0.223%)	**0.584% (Saliva: 0.001%)**	0.061% (Saliva: 0.17%)	0.001% (Saliva: 0.753%)	0.002% (Saliva: 0.002%)	0%	0%
*Treponema Denticola*	0%	0%	0%	0%	0%	0%	0%	**0.146% (Saliva: 0.469%)**	**2.507% (Saliva: 0.012%)**	0.001% (Saliva: 0.07%)	0%	0%	0%	0.001% (Saliva: 0.073%)	0%	0.001% (Saliva: 0.002%)	**0.713% (Saliva: 0.42%)**	0%
*Tannerella Forsythia*	0%	**(Saliva: 0.066%)**	0.001% (Saliva: 0.092%)	0.001% (Saliva: 0.003%)	0%	0%	0.001% (Saliva: 0.017%)	**0.038% (Saliva: 0.17%)**	0.007% (Saliva: 0.011%)	**0.015% (Saliva: 0.046%)**	0%	**0.247% (Saliva: 0.056%)**	0%	**0.016% (Saliva: 0.022%)**	0%	0.002% (Saliva: 0.002%)	**0.733% (Saliva: 0.264%)**	0%
